# Supplementary material for: Integration of Genome-Wide Identification and Transcriptome Analysis of Class III Peroxidases in Paeonia ostii: Insight into Their Roles in Adventitious Roots, Heat Tolerance, and Petal Senescence
Source: Int J Mol Sci. 2024 Nov 12;25(22):12122. doi: 10.3390/ijms252212122 (PMC11593962; doi:10.3390/ijms252212122)
Supplement: Supplementary file 1 [file ijms-25-12122-s001.zip › ijms-3289316-supplementary.pdf]

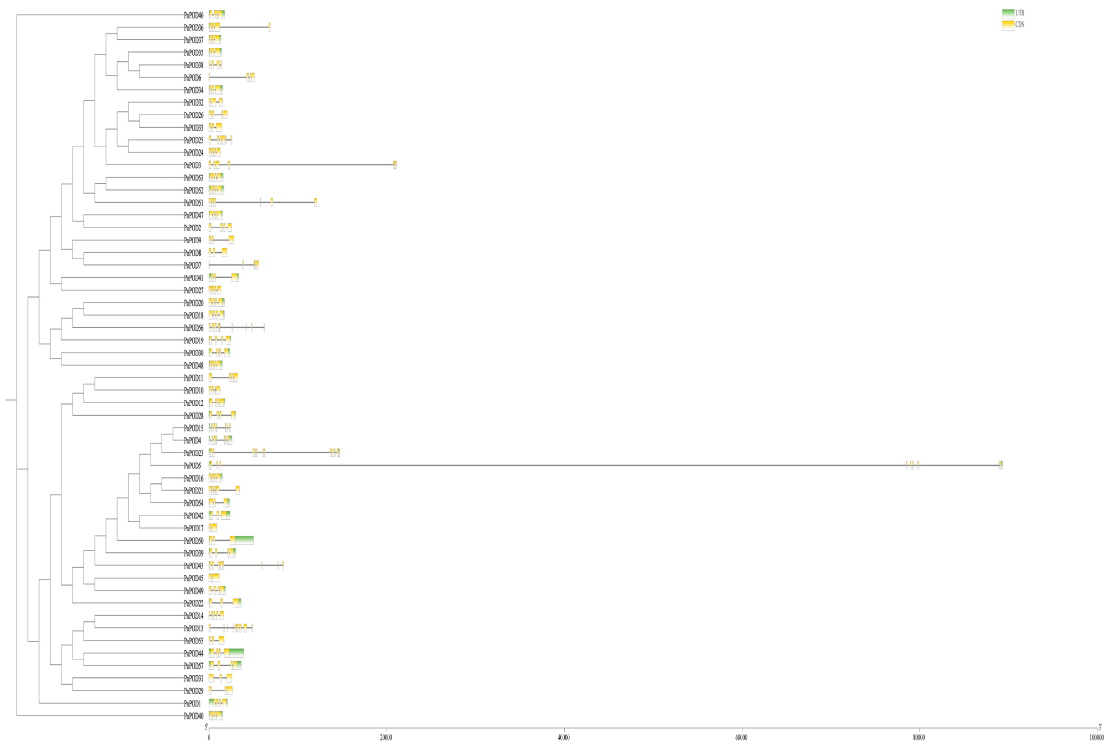

Figure S1. The coding sequences (CDS) and untranslated regions (UTR) for PODs in *P. ostii* are represented by yellow and green boxes, respectively. At the bottom of the figure, the relative position is proportionally displayed based on the kilobase scale.

Supplementary Table S1 Primers used in this study.

Primers Name	Primer sequence (5′-3′)
PoPOD4-F	GGTGCCACAAGGAGCGAT
PoPOD4-R	AGGGTCGGCCAAAAGAGC
PoPOD15-F	CCCCCAGAAGGTCGCTTG
PoPOD15-R	CCAGTGTGTGACCACCGG
PoPOD23-F	GCTGGTCTCGTGAACGCT
PoPOD23-R	TTAGGACCCCCAGCCTCC
PoPOD41-F	CCAGAAGCGTTGCCTCCA
PoPOD41-R	GAGCATGTCCGGGGTGTC
PoPOD55-F	CACCCCTGGAGTGGTTGC
PoPOD55-R	CCTGGGTTGAGTGCAGGG
Actin F	GGTCTATTCTTGCTTCCCTCAG
Actin R	GAACTCACTATCAAACCCTCCAG
